# Resilience-based Islamic program as a promising intervention on diabetes fatigue and health-related quality of life

**DOI:** 10.1371/journal.pone.0273675

**Published:** 2022-09-01

**Authors:** Kusnanto Kusnanto, Hidayat Arifin, Rifky Octavia Pradipta, Gusmaniarti Gusmaniarti, Heri Kuswanto, Agus Setiawan, Bih-O Lee

**Affiliations:** 1 Department of Fundamental Nursing Care, Faculty of Nursing, Universitas Airlangga, Surabaya, Indonesia; 2 Department of Medical and Surgical Nursing, Faculty of Nursing, Universitas Padjadjaran, Bandung, Indonesia; 3 Doctoral Program in School of Nursing, College of Nursing, Taipei Medical University, Taipei, Taiwan; 4 Early Childhood Teacher Education Study Program, Faculty of Teacher Training and Education, Universitas Muhammadiyah Surabaya, Surabaya, Indonesia; 5 Department of Statistics, Institut Teknologi Sepuluh Nopember, Surabaya, Indonesia; 6 Department of Community Health Nursing, Faculty of Nursing, Universitas Indonesia, Depok, Indonesia; 7 College of Nursing, Kaohsiung Medical University, Kaohsiung, Taiwan; The University of the West Indies, JAMAICA

## Abstract

Psychological problems commonly experienced by patients with type 2 diabetes mellitus (T2DM) cause diabetes fatigue conditions that can further worsen the treatment prognosis. We conducted this investigation to determine the effectiveness of a resilience-based Islamic program on diabetes fatigue and health-related quality of life (HRQoL) by measuring the biochemical indicators of T2DM. This was a quasi-experimental study performed from May to August 2021, in which 80 respondents aged 18–64 years diagnosed with T2DM were included through purposive sampling at a male:female sex ratio of 1:1 in the control group and 17:23 in the treatment group. A resilience-based Islamic program (a combination of stress management, mindfulness, prayer, and *dhikr* (the ritual formula of Sufi brotherhood recited devotionally in praise of Allah and as a means of attaining ecstatic experience)) was implemented in the treatment group for six sessions by blended online and offline interventions. Multidimensional Fatigue Inventory-20 and World Health Organization Quality of Life, Brief Form were used to evaluate diabetes fatigue and HRQoL. Blood tests were performed to measure HbA1c, total antioxidant serum, insulin, cholesterol, triglyceride, high-density lipoprotein cholesterol (HDL-c), and low-density lipoprotein cholesterol (LDL-c) levels from baseline to 3 months. Statistical analyses were conducted using paired *t* test, Wilcoxon signed-rank test, independent *t* test, and Mann–Whitney U test. The resilience-based Islamic program had a beneficial impact on the levels of HbA1c (p < 0.001), lipid profile (triglyceride) (p = 0.011), HDL-c (p = 0.01), LDL-c (p < 0.001), total antioxidant serum (p = 0.001), insulin (p < 0.001), diabetes fatigue (p < 0.05), and HRQoL (p < 0.05) in patients of the treatment group. The results of biochemical tests related to T2DM also indicated a reduction in diabetes fatigue and an increase in HRQoL due to the resilience-based Islamic program. Considering that a patient’s resilience to diabetes is an important factor in the management of diabetes fatigue, the resilience-based Islamic program can be applied at public health centers and community levels to increase T2DM resilience.

## Introduction

Diabetes mellitus (DM), a noncommunicable disease (NCD), is a major health problem in Indonesia [1]. Due to its increasing prevalence each year, there is a need for appropriate approaches and interventions to overcome DM. In addition to its known negative impacts on one’s physical health, patients with type 2 DM (T2DM) are highly susceptible to psychological disorders such as worries related to the illness, stress, and burnout from the treatment process, which often lasts for a long time, and diet that must be strictly followed [2–4]. This constitutes fatigue both physically and psychologically, which, in turn, causes diabetes fatigue [5]. This situation can have an impact on the treatment process and goals of DM management.

The major difference between type 1 DM (T1DM) and T2DM is that T1DM is a hereditary illness that frequently manifests in infancy, whereas T2DM is primarily lifestyle-related and manifests over time [6]. In T1DM, the immune system attacks and kills the insulin-producing cells in the pancreas. It has been reported that T1DM affects 8% of all people with diabetes, whereas approximately 90% of people worldwide have T2DM [6]. In Indonesia, the prevalence of DM has been continuously increasing every year. According to the Baseline Health Research of Indonesia, the prevalence of DM among individuals aged >15 years increased by 8.5% in 2018. In women, the prevalence rate increased by 12.7% compared with that 9% increase in men. Data on medicine intake compliance accounted for 11% of all people lost to follow-up to treatment [7]. People with diabetes are two to three times more likely to have depression than those without diabetes. Only 25%–50% of people with T2DM with depression can be diagnosed and treated, whereas the remaining are undiagnosed and thus experience the severity of the condition [8]. This situation of remaining undiagnosed causes 10.37 times greater risk of suffering from diabetes fatigue with a prevalence of 53% [9].

Diabetes fatigue generally occurs due to psychological problems. A previous study showed that it is a multifactorial syndrome of fatigue among patients with DM caused by lifestyle [10] and nutritional, medical, psychological, glycemic/diabetes-related, endocrine, and iatrogenic factors [5]. Diabetes fatigue is often caused by the condition’s complicated process and longevity, which can often be associated with physiological variables (acute hyperglycemia/hypoglycemia, chronic hyperglycemia, glucose variability, and diabetes symptoms), psychological variables (diabetes emotional distress and depressive symptoms), and lifestyle variables (increased body mass index and reduced physical activity) [11]. Therefore, appropriate intervention is required to treat diabetes fatigue. Several previous studies that have examined fatigue-maintaining cognitions, behaviors [12], and psychological interventions [13] can help identify interventions that could mitigate or reduce fatigue. However, those studies are still restricted in their coverage of specific psychiatric issues, indicating the need for further development.

Being an NCD, it is necessary to focus on the prevalence, severity, and psychological effects of DM. The primary challenge in NCD prevention is to develop predictive, preventive, and personalized medicine (PPPM) [14]. To stand the predictiveness of DM, we need the prognosis of treatment algorithm and efficiency, early diagnosis, risk assessment, and innovative screening. Prevention begins with improving public health education, targeted prevention of potential complications, and effective treatment management. Additionally, treatment algorithms tailored to the individual, personalized therapy monitoring and prognosis, and personalized patient profiling are required for personalized medical care [14]. Preventive and personalized medical care can be used as a management approach to minimize the effects of diabetes distress to overcome diabetes fatigue. Therefore, in this study, we developed a resilience-based Islamic program to overcome diabetes fatigue and improve one’s quality of life. This resilience program consisted of stress management and mindfulness, along with spiritual intervention through an Islamic program. Better stress management, mind management through mindfulness, acceptance of DM pain, bounce back, and a spiritual approach to God can be used as a continuous intervention to improve resilient conditions. We aimed to determine the effects of the resilience-based Islamic program in reducing diabetes fatigue and improving quality of life by measuring the biochemical indicators of T2DM.

## Materials and methods

### Study design

We used a quasi-experimental design to demonstrate the causality between an intervention and an outcome [15]. Such a method was used because of the difficulty in randomizing according to subject and location due to restrictions during the coronavirus disease 19 (COVID-19) pandemic.

### Setting and sample

**Fig 1:**
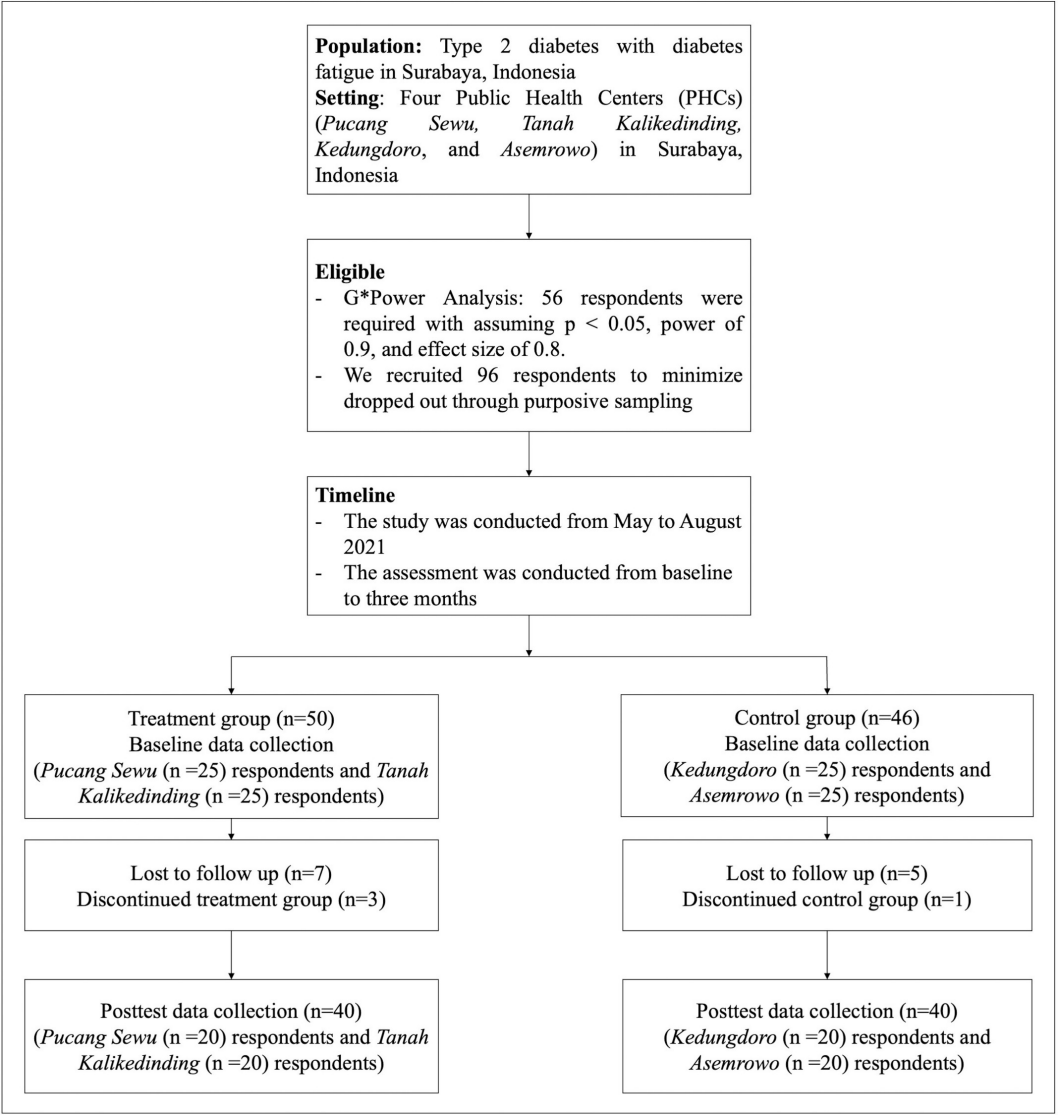
Sample diagram flow chart.

This study was conducted at four public health centers (PHCs), Pucang Sewu, Tanah Kalikedinding, Kedungdoro, and Asemrowo in Surabaya, Indonesia, from May to August 2021. We selected Surabaya as the study location because it is the second-largest city in Indonesia with a high prevalence of T2DM. Locations in the control and intervention groups are widely spread across the city of Surabaya. We categorized Pucang Sewu and Tanah Kalikedinding as the treatment group and Kedungdoro and Asemrowo as the control group based on their location. The location of PHCs was selected as a measure to prevent contact among respondents to reduce bias. The specified population was patients with T2DM (International Classification of Diseases: E11)) in Surabaya city, Indonesia. We conducted a power analysis using G*Power (software used to calculate statistical power) version 3.1.9.6 [16, 17] to determine the minimum sample size required to evaluate the effectiveness of the resilience-based Islamic program. A total of 56 respondents were required, assuming p < 0.05, power 0.9, and effect size 0.8. We obtained a sample of 96 respondents to minimize drop out through purposive sampling and divided them into two groups [treatment (Pucang Sewu = 25 respondents and Tanah Kalikedinding = 25 respondents) and control (Kedungdoro = 25 respondents and Asemrowo = 21 respondents)]. The inclusion criteria of study participants were as follows: (1) must be *compos mentis*, (2) a Muslim to implement prayer and *dhikr* (the ritual formula of Sufi brotherhood recited devotionally in praise of Allah and as a means of attaining ecstatic experience [18]), (3) aged 18–64 years (young adults to older adults who can legally sign the informed concern) and diagnosed with T2DM, (4) with diabetes fatigue condition who have been screened using the Multidimensional Fatigue Inventory (MFI-20) questionnaire, (5) undergoing treatment for T2DM for at least 3 years; (6) ability to write and speak Bahasa (Indonesian national language) and Javanese (local language), and (7) willing and committed to participate in this research until completion. Respondents were excluded if they (1) experienced generalized anxiety disorder and major depressive disorder and have been screened using the Patient Health Questionnaire 4 [19, 20], (2) had a hemoglobin level of <10 mg/dL, and (3) could not participate in the research process until completion. In the treatment group, during the intervention, seven of 50 respondents were lost to follow-up, and three discontinued (two worked in a different city and one moved to a different residence) from the study (Pucang Sewu = 20 respondents and Tanah Kalikedinding = 20 respondents). In the control group, of 46 respondents, five were lost to follow-up, and one discontinued from the study (Kedungdoro = 20 respondents and Asemrowo = 20 respondents). Finally, there were 80 respondents (Fig 1).

### Variables

The independent variable in this study was the resilience-based Islamic program. Resilience for the mind consists of well-being, social connection, and methods to cope, which was developed by Shaun Goodwin [21]. Meanwhile, the Islamic intervention includes the five daily prayers and *dhikr*. The dependent variables included glycated hemoglobin A1c (HbA1c), total antioxidant serum (TAS), lipid profile, cholesterol, triglycerides, high-density lipoprotein cholesterol (HDL-c), low-density lipoprotein cholesterol (LDL-c), insulin, diabetes fatigue, and health-related quality of life (HRQoL).

### Measurements

#### Biochemical measurements

To investigate the biochemical indicators of T2DM, 10 cc of blood was collected from the median cubital vein of each participant. All blood samples were examined at a nongovernment laboratory in Surabaya, Indonesia. The HbA1c level (normal value: <5.7%) [22] was measured using the turbidimetric inhibition immunoassay method. The TAS level (normal value: 1.30–1.77 mmol/L) [23] was measured using colorimetric method and checked using Randox total antioxidant control at a temperature of +37°C. The serum concentrations of total cholesterol (normal value: 200 mg/dL), triglycerides (normal value: 150 mg/dL), HDL-c (normal value: 60 mg/dL), and LDL-c (normal value: 100 mg/dL) were analyzed using the HDL and LDL/VLDL cholesterol assay kit [24]. The insulin level (normal value: 5–25 μU/mL) was measured using RayBio^®^ Human Insulin ELISA Kit [25].

#### Diabetes fatigue

Diabetes fatigue was evaluated using the English version of MFI-20 [26]. We translated the MFI-20 questionnaire to the Indonesian version with the help of an Indonesian linguist at Universitas Airlangga, Indonesia. This questionnaire consists of five dimensions, namely, general fatigue, physical fatigue, mental fatigue, reduced activity, and reduced motivation. Each dimension consists of four items and a 5-point Likert scale (1 referring to no, that is not true, and 5 referring to yes, that is true), with a value range of 4–20 in each dimension. The higher the score of each dimension is, the higher the fatigue. The MFI-20 has demonstrated adequate internal consistency and reliability (Cronbach’s α = 0.831) [27]. Respondents filled in the MFI-20 questionnaire independently; they were helped by the researcher if they could not understand any question.

#### Health-related quality of life

The World Health Organization Quality of Life, Brief Form (WHOQOL-BREF) originally consists of 26 items, but for this study, we used the WHOQOL-BREF Indonesian version [28], which has been confirmed to be reliable and valid across several different populations [29]. For the WHOQOL-BREF, a 5-point Likert scale was used to evaluate participants’ health and well-being. Each item was graded on a scale from 1 to 5, with 1 being the poorest condition and 5 being the best condition. Questions 3, 4, and 26 had a negative value. Therefore, higher ratings indicated higher quality of life. The WHOQOL-BREF also has four domains: physical health (questions 3, 4, 10, 15, 16, 17, and 18), psychological health (questions 5, 6, 7, 11, 19, and 26), social relationship (questions 20, 21, and 22), and environmental quality of life (questions 8, 9, 12, 13, 14, 23, 24, and 25) [30]. Questions 1 and 2 are related to participants’ overall QoL and health in general. The WHOQOL-BREF Indonesian version has been shown to have adequate internal consistency and reliability (Cronbach’s α = 0.880) [31].

### Study procedures

This study was conducted in Indonesia during the COVID-19 pandemic. The assessment was conducted from baseline to 3 months. The intervention process in the treatment group was implemented through a blended learning approach (offline and online using video conferencing media). Meanwhile, the control group was offered an offline intervention when the respondent came for control to the PHC. The two groups in this study were pretested, and 10 cc of blood was collected at the first session and then at the final session. All blood samples and questionnaires were coded by researchers to maintain the privacy of the information of respondents. The intervention was conducted by researchers and PHC nurses. The laboratory personnel with a medical analyst educational background performed blood sampling, which was previously explained in relation to this research. The evaluators (blood collection and questionnaire) were aware of the condition of the two groups. We ensured that they did not have any conflicts of interest with the respondents.

In the treatment group, the researchers applied the resilience-based Islamic program to 40 respondents in two PHCs. The intervention was provided for 3 months with six sessions. Table 1 summarizes the details of the intervention. The resilience-based Islamic program was implemented by providing support to patients with T2DM experiencing diabetes fatigue through stress management (managing stress, anxiety, worry, and panic that caused diabetes fatigue), mindfulness (hopes and expectation; identifying the source of fatigue, distorted thoughts, and moods by understanding anxiety, stress, and how we react; challenging distorted thoughts and mood; and setting goals and challenges), and Islamic intervention by regularly performing five daily prayers and *dhikr*. Respondents were also given a module, which was developed by the researcher, to teach them how to manage diabetes fatigue. The module contained information related to DM, diabetes fatigue, and spiritual approach, as well as a checklist for the five daily prayers and *dhikr*. In the third and fourth sessions, the intervention was implemented online by video conferencing due to the increasing number of COVID-19 cases and the ensuing restrictions (i.e., no gatherings, making it impossible to perform the session in person). In addition to the session, the intervention was provided offline in consideration of everyone’s safety and compliance with the COVID-19 health protocol laid out by the Indonesian government. The required duration for each session was 45–60 min.

**Table 1:** Overview of the resilience based Islamic program.

Session	Intervention
**1**	Hopes and expectation. Looking at how diabetes fatigue and stress affects thoughts feelings, physical wellbeing, and behavior. Islamic strengthen through five times prayers and dzikr.
**2**	Identifying source of fatigue, distorted thoughts, and moods by understanding anxiety, stress and how we react.
**3**	Challenging distorted thoughts and mood. How we can limit ourselves though habitual negative thoughts and moods.
**4**	Managing stress, anxiety, worry, and panic that caused diabetes fatigue. Strengthen though acceptance, controlling panic, learning how to relax, and importance of doing so.
**5**	Setting goals and challenges. Understanding passive anger and resistance. Learning about comfort zone and panic zones.
**6**	Reviewing learning and planning for the future.
**Throughout the course**	A different relaxation technique is introduced in each session, including techniques based on mindfulness and Islamic intervention.

The control group consisted of 40 respondents from two PHCs. These respondents were provided an intervention according to the health program for the management of NCDs, specifically DM at the PHC. The program was implemented once a month when the patient came for control to the PHC. The intervention was conducted for 3 months, with one session every month. In the first month, the respondents received health education interventions about diabetes fatigue. The next session was about physical activity through DM exercise, and the final session provided spiritual intervention such as religious lectures. Each intervention was conducted for 45–60 min.

### Data analysis

Statistical analyses were conducted using Stata version 16.1. Descriptive statistics such as numbers, percentages, mean, and standard deviation were used to represent the descriptive characteristics of the respondents in both groups. Fisher’s exact test was used to evaluate the probability of most of the differences between groups. The Kolmogorov–Smirnov test was used to evaluate the normality of the distribution of variables. As the data were skewed, we also used the median and interquartile range to represent the results. The paired *t* test and independent *t* test were conducted in the case of normal distribution, and the Wilcoxon signed-rank test and Mann–Whitney U test were conducted in the case of nonnormal distribution. The paired *t* test and Wilcoxon signed-rank test were also performed to analyze the preintervention and postintervention test values of each group. The independent *t* test and Mann–Whitney U test were performed to compare both groups. Cohen’s d coefficient with a mean of posttest data was used to describe the effect size between treatment and control groups. The Cohen’s d coefficient indicates the direction of the effect. Negative effect size indicates that the effect decreases the mean, and a positive effect size indicates that the effect increases the mean. Next, we categorized the effect into very small (0.01), small (0.2), medium (0.5), large (0.8), very large (1.2), and huge (2.0) [32]. Results were expressed using a 95% confidence interval and a level significance of p < 0.05, with a two-tailed statistical test.

### Ethical consideration

This research was approved by the Health Research of Ethics Commission, Faculty of Nursing, Universitas Airlangga, Surabaya, Indonesia, with No: 2092-KEP. Participants were required to provide their written consent to participate free of coercion. They could withdraw from the study without giving any reason and with no impact on their health care.

## Results

Of the 80 respondents, the treatment group primarily consisted of women (57.5%), whereas the control group had an equal number of men and women. Half of the respondents were aged 35–44 years in the treatment group, and almost half of them in the control group were aged 45–54 years (47.5%). In both groups, the majority of respondents were married, worked in nongovernment sectors, lived in urban areas, and had secondary education. There were more respondents with high educational levels in the treatment group than in the control group. The remaining data were homogeneous (p > 0.05) (Table 2).

**Table 2:** Respondent’s characteristic demography.

Characteristic	Group				*p*
	Treatment (n = 40)		Control (n = 40)		
	n	%	n	%	
**Sex**					
**Male**	17	42.5	20	50	0.200*
**Female**	23	57.5	20	50	
**Age**					
**25–34**	5	12.5	2	5	0.603*
**35–44**	20	50	10	25	
**45–54**	7	17	19	47.5	
**54–65**	8	20	9	22.5	
**Marital status**					
**Married**	28	70	36	90	0.642*
**Unmarried**	10	25	-	-	
**Widow/widower**	2	5	4	10	
**Education**					
**Primary education**	4	10	15	37.5	0.638*
**Secondary education**	19	47.5	24	60	
**High education**	17	42.5	1	2.5	
**Occupation**					
**Government**	18	45	13	32.5	> 0.999*
**Non-government**	22	55	27	67.5	
**Residence**					
**Urban**	23	57.5	21	52.5	0.062*
**Rural**	17	42.5	19	47.5	

*Fisher’s exact test; homogeneity p > 0.05

Overall, the resilience-based Islamic intervention exerted an impact on the levels of HbA1c, lipid profile, TAS, insulin, diabetes fatigue, and HRQoL among patients with T2DM. However, as shown in Table 3, only in the control group did the variable of HRQoL in the dimension of physical health and environmental quality of life show a significant difference between preintervention and postintervention compared with the treatment group. In the treatment group, only the variable cholesterol showed no change between preintervention and postintervention. Regarding the HbA1c level, it showed a decrease in the mean, minimum, and maximum values in the treatment group but an increase in the control group. The statistical analysis revealed a significant difference in terms of preintervention and postintervention tests in the treatment group (p < 0.001) but not in the control group. Meanwhile, the independent *t* test revealed a significant difference between the treatment and control groups based on posttest results (p = 0.004). The TAS levels showed an increase in the mean value before and after intervention in the treatment group but a decrease in the control group. A significant difference was observed between the preintervention and postintervention test results in the treatment group (p = 0.001) but not in the control group (p = 0.216). The TAS levels in both groups also showed a significant difference based on posttest results (p < 0.001) (Table 3).

**Table 3:** The distribution and analysis of HbA1c, TAS, lipid profile, insulin, diabetes fatigue, and health related quality of life.

Group	n	Pre-test			Post-test			Difference	*95% CI*	*d*	*p* *	*p* **
		Mean ± SD	Min—Max	Median ± IQR	Mean ± SD	Min—Max	Median ± IQR	Mean ± SD				
**HbA1c (%)**												
**Treatment**	40	6.72 ± 0.67	5.2–7.9	6.85 ± 0.84	6.16 ± 0.77	4.8–7.5	6.25 ± 1.5	-0.56 ± 0.1	5.91–6.41	- 0.65	<0.001^a^	0.004^c^
**Control**	40	6.58 ± 0.73	5.5–7.8	6.4 ± 1.3	6.62 ± 0.62	5.6–7.7	6.5 ± 1.0	0.04 ± -0.11	6.42–6.82		0.377^a^	
**TAS (mmol/L)**												
**Treatment**	40	1.55 ± 0.54	0 .6–2.5	1.55 ± 0.9	1.79 ± 0.49	0.9–2.7	1.9 ± 0.59	0.24 ± -0.05	1.63–1.95	0.80	0.001^a^	<0.001^c^
**Control**	40	1.44 ± 0.58	0.5–2.5	1.25 ± 0.95	1.39 ± 0.50	0.7–2.6	1.15 ± 0.95	-0.05 ± -0.08	1.22–1.55		0.216^a^	
**Lipid Profile**												
***Cholesterol (mg/dL)***												
**Treatment**	40	211.57 ± 57.85	112–350	206.0 ± 63.0	200.17 ± 44.05	112–289	200.0 ± 50.5	-11.4 ± -13.8	186.08–214.26	-0.45	0.099^a^	0.044^c^
**Control**	40	217.25 ± 53.27	112–372	220.0 ± 53.0	220.4 ± 44.28	150–321	220.0 ± 60.0	3.15 ± -8.99	206.23–234.56		0.649^a^	
***Triglyceride (mg/dL)***												
**Treatment**	40	163.65 ± 46.31	85–273	151.5 ± 73.0	137.82 ± 27.13	89–190	132.0 ± 32.5	-25.83 ± -19.18	129.14–146.50	-0.09	0.011^a^	<0.001^c^
**Control**	40	168.27 ± 32.86	89–270	175.0 ± 42.0	166.77 ± 31.62	120–250	170.0 ± 48	-1.5 ± -1.24	156.65–176.89		0.64^a^	
***HDL-c (mg/dL)***												
**Treatment**	40	57.2 ± 13.2	34–80	54.5 ± 21.0	67.17 ± 11.16	43–88	67.5 ± 16.0	9.97 ± -2.04	63.60–70.74	1.07	0.01^a^	<0.001^c^
**Control**	40	56.17 ±13.62	34–87	54.5 ± 22.0	55.05 ± 11.41	39–77	55.5 ± 17.5	-1.12 ± -2.21	51.39–58.70		0.339^a^	
***LDL-c (mg/dL)***												
**Treatment**	40	116.55 ± 20.53	80–154	120.0 ± 30.0	105.35 ± 19.39	80–150	100.0 ± 25.0	-11.2 ± -1.14	99.14–111.55	- 0.56	<0.001^a^	0.014^c^
**Control**	40	113.87 ± 20.16	80–150	116.0 ± 24.5	116.2 ± 19.22	88–150	115.0 ± 30.0	2.33 ± -0.94	110.05–122.34		0.272^a^	
**Insulin (μU/mL)**												
**Treatment**	40	6.31 ± 1.13	4.0–8.9	6.45 ± 1.4	6.96 ± 1.19	4.2–9.1	7.0 ± 1.9	0.65 ± 0.06	6.57–7.34	1.29	<0.001^b^	<0.001^d^
**Control**	40	5.54 ± 1.05	3.4–7.8	5.5± 1.4	5.48 ± 1.09	3.1–7.4	5.65 ± 1.0	-0.06 ± 0.04	5.13–5.82		0.954^b^	
**Diabetes Fatigue**												
***General Fatigue***												
**Treatment**	40	13.35 ± 1.77	9.0–17.0	13.0 ± 3.0	10.1 ± 2.14	6.0–15.0	10.0 ± 3.5	-3.25 ± 0.37	9.41–10.78	- 0.92	0.022^b^	0.001^d^
**Control**	40	11.95 ± 1.73	9.0–17.0	12.0 ± 3.0	12.05 ± 2.06	8.0–17.0	12.0 ± 4.0	0.1 ± 0.33	11.39–12.70		0.681^b^	
***Physical Fatigue***												
**Treatment**	40	12.07 ± 1.84	9.0–16.0	12.0 ± 2.0	9.62 ± 1.97	5.0–15.0	9.0 ± 3.0	-2.45 ± 0.13	8.99–10.25	- 1.22	<0.001^a^	<0.001^c^
**Control**	40	12.12 ± 2.23	8.0–17.0	12.0 ± 3.5	12.0 ± 1.92	8.0–16.0	12.0 ± 3.0	-0.12 ± -0.31	11.38–12.61		0.49^a^	
***Reduced Activity***												
**Treatment**	40	12.2 ± 2.28	8.0–17.0	12.0 ± 4.0	9.65 ± 2.63	5.0–16.0	10.0 ± 3.0	-2.55 ± 0.35	8.80–10.49	- 0.88	<0.001^a^	<0.001^c^
**Control**	40	11.25 ± 1.79	8.0–17.0	11.0 ± 2.0	11.6 ± 1.69	8.0–15.0	11.0 ± 3.0	0.35 ± -0.1	11.05–12.1		0.132^a^	
***Reduced Motivation***												
**Treatment**	40	11.97 ± 2.20	8.0–16.0	12.0 ± 4.0	9.52 ± 2.39	5.0–14.0	10.0 ± 3.5	-2.45 ± 0.19	8.75–10.29	- 1.07	<0.001^a^	<0.001^c^
**Control**	40	11.65 ± 2.48	8.0–17.0	12.0 ± 4.0	11.87 ± 1.98	8.0–15.0	12.0 ± 3.0	0.22 ± -0.5	11.23–12.51		0.346^a^	
***Mental Fatigue***												
**Treatment**	40	12.0 ± 2.16	8.0–16.0	12.0 ± 4.0	9.7 ± 2.37	6.0–15.0	9.5 ± 3.5	-2.3 ± 0.21	8.93–10.46	- 1.20	0.024^a^	<0.001^c^
**Control**	40	12.32 ± 2.78	8.0–17.0	12.0 ± 4.0	12.25 ± 1.83	8.0–15.0	12.5 ± 4.0	-0.07 ± -0.95	11.66–12.83		0.783^a^	
**HRQoL**												
***Physical Health***												
**Treatment**	40	74.42 ± 11	56–94	75.0 ± 17.5	90.42 ± 13.45	56–100	100.0 ± 19.0	16 ± 2.45	86.12–94.72	1.84	<0.001^a^	<0.001^c^
**Control**	40	73.95 ± 12.64	56–100	72.0 ± 18	69.15 ± 9.22	56–88	69.0 ± 12.0	-4.8 ± -3.45	66.19–72.10		0.011^a^	
***Psychological***												
**Treatment**	40	77.87 ± 13.82	56–100	78.0 ± 25.0	96.62 ± 7.28	63–100	100.0 ± 6.0	18.75 ± -6.54	94.29–98.95	3.23	<0.001^a^	<0.001^c^
**Control**	40	70.45 ± 9.37	56–94	69.0 ± 15.0	69.05 ± 9.6	56–93	69.0 ± 12.0	-1.4 ± 0.23	65.97–72.12		0.094^a^	
***Social Relationships***												
**Treatment**	40	78.6 ± 13.26	56–100	78.0 ± 19.0	96.25 ± 8.0	75–100	100.0 ± 0.0	17.65 ± -5.26	93.68–98.81	1.78	0.001^a^	<0.001^c^
**Control**	40	77.07 ± 12.18	56–100	75.0 ± 19.0	77.37 ± 12.67	56–100	75.0 ± 19.0	0.3 ± 0.49	73.32–81.42		0.159^a^	
***Environmental quality of life***												
**Treatment**	40	79.67 ± 14.26	44–100	81.0 ± 22.0	98.2 ± 3.38	88–100	100.0 ± 3.0	18.53 ± -10.88	97.11–99.28	2.12	<0.001^a^	<0.001^c^
**Control**	40	73.92 ± 11.54	56–94	78.0 ± 18.0	76.1 ± 14.3	56–100	75.0 ± 25.0	2.18 ± 2.76	71.52–80.67		0.025^a^	

HbA1c: Glycated Hemoglobin A1c; TAS: Total Antioxidant Serum; HDL-c: High-Density Lipoprotein Cholesterol; LDL-c: Low-Density Lipoprotein Cholesterol; HRQoL: Health Related Quality of Life; SD: Standard Deviation; CI: Confident Interval; d: Cohen’s d coefficient; interquartile range (IQR)

Significant value: p<0.05

p*: intragroup comparison

p**: intergroup comparison

^a^Paired t-test

^b^Wilcoxon Signed Rank Test

^c^Independent t-test

^d^Mann-Whitney U-test

Regarding the lipid profile of cholesterol, triglyceride, HDL-c, and LDL-c levels, the mean, minimum, and maximum cholesterol levels remained stable, but the LDL-c levels were decreased. However, the HDL-c levels were increased and triglyceride levels were decreased. The results of paired *t* test in the treatment group showed that triglyceride (p = 0.011), HDL-c (p = 0.01), and LDL-c (p < 0.001) levels exhibited significant differences between preintervention and postintervention tests besides cholesterol levels (p = 0.099). The independent *t* test of cholesterol (p = 0.044), triglyceride (p < 0.001), HDL-c (p < 0.001), and LDL-c (p = 0.014) levels revealed significant differences between the treatment and control groups based on posttest results. We also found that insulin levels were increased after the intervention (p < 0.001). The Mann–Whitney U test revealed a significant difference between the treatment and control groups based on posttest results (p < 0.001) (Table 3).

We analyzed diabetes fatigue from five dimensions of fatigue. The statistical analysis revealed that these five dimensions exhibited a decrease in terms of mean, minimum, and maximum values between preintervention and postintervention tests. We found that general fatigue (p = 0.022), physical fatigue (p < 0.001), reduced activity (p < 0.001), reduced motivation (p < 0.001), and mental health (p < 0.001) can significantly differ after the intervention in the treatment group but not in the control group. Diabetes fatigue also showed a significant difference in both groups based on posttest results. We analyzed HRQoL in five dimensions, which showed an increase in terms of mean values between preintervention and postintervention tests. The results of the paired *t* test revealed that physical health, psychological health, social relationships, and environmental quality of life showed significant differences in the treatment group (p < 0.001), whereas only physical health and environmental quality of life were significantly different in the control group (p < 0.05). The results of the independent *t* test indicated a statistically significant difference between the two groups based on posttest results (p < 0.001) (Table 3).

## Discussion

This study explored the effectiveness of the resilience-based Islamic program implemented by nurses on patients with diabetes fatigue and determined the changes in HRQoL after the intervention. Furthermore, the biochemical indicators of T2DM such as HbA1c, TAS, insulin, and lipid profiles were measured. The resilience-based Islamic program was implemented because most Indonesians are Muslim.

Diabetes fatigue is closely associated with distress conditions and psychological changes in patients with DM [33]. This can have an impact on the levels of HbA1c [34], TAS, insulin [35], and lipid profiles [36, 37]. A condition of less-than-normal insulin levels can have an impact on glycolysis, decrease the rate of adenosine diphosphatase phosphorylation, and slow down adenosine triphosphatase resistance, which could, in turn, lead to diabetes fatigue. Biochemical conditions are highly sensitive to psychological conditions [5]. The inability to manage diabetes and psychological conditions can have an impact on diabetes fatigue [5]; it can also affect TAS, which subsequently increases the likelihood of oxidative stress and the risk of developing foot ulcers [38].

Improving the psychological conditions of patients with DM can have an impact on physiological biomarkers [39]. Several previous studies have demonstrated that improved psychological disposition can provide a sense of pleasure and a calmer feeling that can trigger the secretion of endorphin and serotonin hormones that influence HbA1c and blood sugar levels [40] and lipid profiles [41]. The resilience-based Islamic program provides interventions to manage stress as well as acceptance of DM disease [4]. This certainly has a positive impact that can be evaluated through changes in physiological biomarkers.

Patients with DM are extremely susceptible to experiencing psychological disorders due to various factors such as the need to undergo therapy and follow a long-term diet plan [42]. Deteriorating conditions can result in diabetes fatigue, wherein patients experience fatigue both physically and psychologically in terms of continuing the treatment [5]. In the present study, diabetes fatigue was examined using the five dimensions of fatigue, i.e., general fatigue, physical fatigue, reduced activity, reduced motivation, and mental fatigue. Based on these dimensions, patients with DM who experience fatigue can suffer from poor health conditions and subsequent treatment. In this case, it is necessary to implement a psychological approach to manage diabetes fatigue. The resilience-based Islamic program led to a significant change in fatigue in all dimensions, which is because the interventions that focus on stress management, mindfulness, and spirituality can increase sincerity and acceptance of DM conditions. Patients who can accept the illness they are suffering from will find it easier to undergo treatment, which can consequently improve the prognosis of their disease [43]. Previous studies have demonstrated that patients’ psychological well-being improves as their resilience increases in the face of chronic illness and disease [44, 45]. Additionally, patients with diabetes who are already resilient can improve their ability to self-care and help others [4].

Besides the fatigue problems experienced by patients with DM, improving the quality of life remains a concern. This is because fatigue conditions can negatively impact one’s quality of life and health [46, 47]. When fatigue conditions decrease, the quality of life may improve, which can be observed from the dimensions of physical health, psychological and social relationships, and environmental quality of life. The resilience-based Islamic program has not only improved the fatigue conditions of patients with DM but has also increased their HRQoL. This is because the improvement of fatigue conditions is followed by an improvement in the quality of life [48]. Previous research has demonstrated that resilient conditions can provide improvement under illness conditions where the focus of intervention is on psychological improvement [49, 50]. The integration of physical and psychological care is an effective intervention to improve the quality of life and quality of care [51]. Additionally, the spiritual approach is an important intervention to increase acceptance.

This study revealed a significant difference in all variables between the treatment and control groups. However, the respondents in the treatment group were younger, more educated, have fewer marriages, and have stable government jobs compared with those in the control group, which can influence the intervention and the study result. Recent studies have shown that respondents of the productive age and with stable economic status have sufficient energy, agility, and capabilities to receive and follow the intervention appropriately [52–54].

### Strength and limitation

The resilience-based Islamic program can be a promising intervention in the future to overcome diabetes fatigue. It can be implemented as a program in the PHC or hospital setting as a complementary intervention among subjects with T2DM. It can be easily implemented using a book guide. This investigation followed a quasi-experimental design, indicating that it has the limitation of not being randomized, due to which a causal association between an intervention and an outcome cannot be concluded [55]. However, this limitation was due to the COVID-19 pandemic, during which we faced numerous restrictions from the government to have direct contact with respondents for performing several equipment-related and standard operational procedures. Moreover, we had limited effective work hours which reduced the researcher’s activity to perform the study. In the future, time-series measurements can be performed to obtain information regarding the effectiveness of the intervention regularly. Finally, the study results may be affected by the restrictions implemented due to the COVID-19 pandemic.

## Conclusion

The resilience-based Islamic program can be used as a promising intervention to overcome diabetes fatigue and improve HRQoL and DM-associated biomarkers. This program provides interventions through stress management, mindfulness, and spiritual approaches through five daily prayers and *dhikr*, which can have an impact on providing a feeling of calmness and acceptance of DM. Moreover, this intervention might help patients with DM to adhere to treatment so that their blood sugar levels can be controlled. The long-term goal is to improve self-care and quality of life. This program can be easily implemented by nurses at the PHC to help patients with diabetes fatigue conditions and become a reference and referral intervention. Further researchers can develop this program using other religious and cultural approaches.

## Supporting information

S1 Raw data (XLSX)
